# The Barnes Method of Brushing Teeth*Extract from *Dental Summary* for June, 1921.

**Published:** 1922-01

**Authors:** Henry Barnes

**Affiliations:** Cleveland


### THE BARNES METHOD OF BRUSHING TEETH*

BY HENRY BARNES, CLEVELAND

The Barnes method of brushing the teeth is effective because of the shape of the brush and the technic used. The brush is a very narrow one, and it is applied by placing the ends of the bristles on the gums and rotating the brush on its handle into the interproximal spaces. The chisel, squeegee and massage action of the bristles necessitated the lingual brush, with practically the same bristle action on lingual contours. The lingual brush was then evolved with a short transverse row of bristles pointing from a sharp curved handle end, and essentially paralleling the handle. This brush has also a curve at the other end to facilitate holding.

* Extract from *Dental Summary* for June, 1921.

The lingual pick brush is used by holding the curved end of the handle with the thumb and forefinger and by a jiggling action upon the gums. This will cleanse all the lingual contours including the condyles. This may be compared to a raking action except that the bristles parallel the handle. This brush may be of one row of stiff bristles or of two rows which converge at their ends which is practically a one-row brush.

### TO START CONTOUR BRUSHING

Take the brush kept in water or soap water and soften in hot water. Hot water is preferable from spigot as it will be needed to clean the brush frequently during each brushing. Dipping brush into a glass of water is filthy because it is literally trying to cleanse the mouth with its own debris.

The characteristic positions of teeth and their spaces is vertical. Therefore the essential movement to produce the proper action of the brush must be vertical. There also must be such a brush-hold as will permit a compensating action of the brush, hand and wrist to follow the contour of the dental arch. This brush-hold must be flexible, but the left side is brushed with the brush held as though it were a pen or pencil. The bristles point down on upper arch and up on lower arch.

A pivotal hold is made with the tips of thumb and forefinger on handle close to bristles, other fingers acting as guides, motors and brakes. For a right-handed person, surfaces from which it is chiseled and carried away by the bristles. The sides of the bristle clusters also act as a massage in the interdental spaces. Also they serve to give the concave shape to their gum surfaces for the proper sluicing of food during mastication.

The motion should be comparatively slow to give the bristles time to flex and follow contours from gum to tooth and interdental contour. The slow flexation of the bristle chisels into the interdental spaces with slight additional pressure down, in, through and back repeatedly, has been well expressed by Dr. Hartzell with the term "jiggle." The writer has used and taught this action since 1911.

To brush the left side the brush is turned in the pivotal fingers and brought around so that it now occupies a position ninety degrees from that of the penhold. Bristles pointing towards you.

Lay the side of the brush high on the upper gum bristles pointing down and flex to an angle of about thirty degrees with the gum. Then with an arm and shoulder motion, stroke down and in with a little more pressure to force in, and also with the jiggle to thoroughly cleanse. This picks the debris from the interdental spaces.

This causes the flexible bristles to act as flexible chisels which scour and massage and squeegee the gums. Debris is squeegeed from the gums and from under the free margins of the gums and from pyorrhea pockets on the tooth food during the act of mastication.

To insure a correct technic and prevent conscientious abuse at home, there should be as frequent visits to the office as may be found necessary for inspection and correction, to correct faulty technic.

The patient may believe that he is following the correct technic whereas the office observation may prove otherwise.

### SHORT GUMS

When the gums are short and the frenum high on the lower jaw, a small soft-bristle brush of two or more rows is recommended, using the rolling method to massage the gums and following by the one-row bristle brush to cleanse the spaces.

Very irregular teeth will require a little deviation from the action used where regularity exists.

The gums will form themselves about the teeth in the direction of the pressure applied by the brush in the act of cleansing. Extreme pressures are to be avoided if natural approximation of gum restoration is desired.

The brush should be held by the rigid fingertips, not in the palm of the hand.

In all the brushings the bristles do not leave the surface but are carried down and up on the upper jaw, and up and down on the lower jaw; the brushes sliding back have only a gentle massaging effect without injury to tissue, soft or hard.

The brush should be rinsed frequently in running hot water to cleanse, and bearable hot water should be used to squiggle within the mouth to dislodge small particles of debris picked out in brushing.

Forcing the bristles into the interdental spaces at right angles to the surface will cause the gums to be flattened on their interdental apices. These gums may be healthy but it will not afford the natural sluice-way to deflect the debris from the interdental spaces.

### INTERDENTAL BRUSHING

Interdental brushing is a limited term and applies only to the interdental spaces, whereas contour means this and much more.

### GINGIVAL PYORRHEA

Gingival pyorrhea is that pyorrhea which commences as gingivitis followed by spongy gums and finally pockets within the alveoli. This may be caused by non-brushing, faulty brushing, malocclusion or other injuries influenced by diet and systemic conditions.

Filth is a prolific cause of pyorrhea. Dr. T. B. Hartzell has very appropriately called this filth bacterial filth or mold.

Pyorrhea is impossible in a mouth cleansed by a method as given. Existing pyorrhea will in many cases be eliminated if this method is persistently followed.

